# Bridging Discovery, Mechanism, and Application: An Integrated Strategy for Bioactive Natural Products

**DOI:** 10.3390/molecules31122001

**Published:** 2026-06-08

**Authors:** Tao Liu, Clementina Manera, Francesca Gado

**Affiliations:** 1 School of Basic Medical Sciences, Guangdong Pharmaceutical University, Guangzhou 510006, China; 2 Department of Pharmacy, University of Pisa, 56126 Pisa, Italy; 3 Department of Drug Sciences, University of Pavia, 27100 Pavia, Italy

## 1. Introduction

Natural products remain one of the most reliable starting points for new medicines, agrochemicals, and functional ingredients. Paclitaxel, artemisinin, the statins, and morphine show that molecules refined by evolution often occupy regions of chemical space that synthetic libraries reach only with difficulty. Their reach extends well beyond the clinic, into crop protection, food preservation, and cosmetics, and the same structural richness that makes them valuable also makes them hard to handle: the compounds of interest are usually minor components of crude mixtures, and isolating, identifying, and evaluating them has historically been a primary rate-limiting step. These molecules are no less valuable; what has changed is how we find, interpret, and supply them. Over the past few years, the field has moved away from a largely linear pipeline of extraction, bioassay-guided fractionation, and structure elucidation toward workflows in which chemistry, biology, and computation inform one another from the start [1].

Discovery has shifted most visibly. Bioassay-guided isolation repeatedly rediscovers known scaffolds, which wastes effort and discourages the screening of complex extracts. High-resolution tandem mass spectrometry combined with molecular networking now lets researchers map the chemical content of a crude sample and recognize known compounds before any preparative work begins, while genome mining reads biosynthetic gene clusters directly from sequence data to predict what an organism can make, including the many pathways that stay silent under laboratory conditions [2,3,4]. Curated open resources such as the Natural Products Atlas and COCONUT have made this kind of dereplication routine, and machine-learning models increasingly help annotate spectra and prioritize which features in a dataset are worth chasing [5,6,7]. Campaigns can now focus resources on genuinely new chemistry rather than on confirming the familiar. Because much of this analysis runs on shared open data and software, it also lowers the barrier for laboratories that lack large isolation facilities. Even with these tools, a large share of the features in any untargeted dataset still cannot be matched to a known structure, and narrowing that unknown fraction remains one of the field’s central problems.

Evaluation has changed just as much. Phenotypic assays tell us that an extract or a compound does something, but they rarely tell us how, and a mechanism inferred long after the activity was first seen is easy to get wrong. Chemoproteomics and the wider integration of multi-omics data now allow investigators to identify the protein targets and pathways that a natural product engages inside living cells, without relying on guesswork after the fact [8,9]. Methods such as activity-based protein profiling and thermal proteome profiling can read target engagement across thousands of proteins at once. Consequently, target identification is no longer a separate, downstream task; it is becoming part of how activity is characterized in the first place. Knowing the target this early changes how a programme is prioritized, permitting counter-screening for selectivity and providing a clearer view of safety liabilities before substantial resources are invested. When these readouts are paired with more faithful biological models, including patient-derived organoids that preserve tissue architecture and cellular heterogeneity, mechanism and translational relevance can be assessed in the same experiment [10].

Supply has changed too. Many of the most interesting natural products occur at trace levels in organisms that are slow-growing, protected, or impossible to culture, and total synthesis is often impractical at scale. Synthetic biology and heterologous expression offer a way around this constraint by reconstructing biosynthetic pathways in tractable microbial hosts, turning a scarce extract into a fermentation product; engineered yeast and bacteria already supply terpenoid, alkaloid, and polyketide scaffolds that were once available only from their native sources [11]. Beyond securing material for further study, this route supports greener manufacturing and gives medicinal chemists a renewable platform on which to diversify a scaffold. Automated design–build–test–learn cycles in dedicated biofoundries are steadily shortening the path from a predicted pathway to a strain that actually makes the molecule. Discovery, evaluation, and supply increasingly run as an iterative loop rather than as separate steps.

None of this displaces the analytical backbone of the discipline. High-field NMR, high-resolution mass spectrometry, and, increasingly, spatial and single-cell readouts remain essential for assigning structures and locating compounds within tissues. The contributions to this Special Issue reflect both this established toolkit and newer methods that combine several kinds of readout.

This Special Issue, the second edition of Bioactive Compounds from Natural Sources: Discovery, Evaluation and Applications, brings together ten contributions that speak to these three themes. The collection comprises nine research articles and one review, and the work ranges from microbial and plant chemistry to peptide synthesis, polysaccharide pharmacology, and food-grade delivery systems. Their source material is correspondingly diverse, spanning terrestrial plants, marine algae, cyanobacteria, and microbial fraction libraries, and the contributing teams are based across Europe, Asia, Africa, and the Americas. Several of the studies also build on byproducts or underused biomass, work that links discovery with sustainability. In the commentary that follows, we group the papers by what they contribute to discovery, evaluation, and application rather than by their order of publication.

## 2. Discovery and Dereplication in Complex Matrices

The dereplication problem is addressed directly by Brittin and co-workers (Contribution 1), who combined liquid chromatography–tandem mass spectrometry with yeast chemical genomics to screen more than 40,000 natural product fractions against *Candida albicans* and the drug-resistant species *C. auris* and *C. glabrata*. Among 450 active fractions, the combination of orthogonal readouts marked known chemotypes and mechanisms of action more effectively than individual methods, enabling the exclusion of nuisance compounds prior to isolation efforts. Given rising antifungal resistance, the study offers a workable approach for directing discovery toward the fractions most likely to contain something new.

Duarte and colleagues (Contribution 2) show how chemical profiling and computation can work together on plant material. Essential oils from three *Piper* species were characterized by GC-MS after microwave-assisted hydrodistillation, yielding eighty mostly terpenoid constituents with measurable activity against the cocoa pathogens *Moniliophthora roreri* and *Phytophthora palmivora* that approached the performance of commercial fungicides. Through cheminformatic analysis of lipophilicity (average LogP 3.4), polar surface area (TPSA 11.5 Å^2^), and shared substructures, the authors discussed a potential membrane-disrupting mechanism, presenting these oils as defined structural scaffolds for sustainable crop protection.

A complementary view of plant chemistry comes from Raal and colleagues (Contribution 3), who profiled the polyphenols of raspberry (*Rubus idaeus*) stems, a byproduct of fruit production that is usually discarded. HPLC-MS resolved sixty-two compounds, with protocatechuic acid pentoside dominating across cultivated, garden, and wild samples. The study also tracked how polyphenol content varied with season and with position along the stem, leading to a concrete recommendation to harvest the upper third. It is a useful reminder that careful analytical work on agricultural waste can identify renewable and inexpensive sources of bioactive material.

## 3. From Activity to Mechanism: Evaluating Bioactivity and Targets

Moving from what compounds are present to how they act, three papers center on evaluation. Guo and co-workers (Contribution 4) used the marine cyclopeptide galaxamide as a scaffold for medicinal chemistry, preparing twenty-six analogues through a “3 + 2” strategy that varied the number and position of its D-leucine residues. Screening against the A549 and K562 cancer lines alongside a non-tumor cell line provided a defined structure–activity relationship, with the analogue Gala04 reaching an IC_50_ of 4.2 µM against K562 and showing useful selectivity. The work converts an unusual natural skeleton into a tractable series that medicinal chemists can continue to optimize. Cyclic peptides can engage targets that conventional small molecules miss, so a synthetically accessible marine scaffold of this kind is worth further study.

Zhang and colleagues (Contribution 5) characterized a polysaccharide, NJSPd-1 (molecular weight 18,545 Da), from fermented noni (*Morinda citrifolia*) juice and investigated its protective effects in a cell model of diabetic oxidative stress. After establishing the molecular weight and monosaccharide composition, the authors showed that, in high-glucose-treated HepG2 cells, the polysaccharide acted through the Keap1–Nrf2/HO-1/NQO1 axis, linking a defined structure to a recognizable antioxidant signaling pathway. This kind of structure-to-pathway mapping is what is needed to move polysaccharide pharmacology beyond purely descriptive reports of activity. Noni juice is already consumed widely as a health beverage, so identifying a defined active polysaccharide with a known mechanism gives that traditional use a firmer scientific footing.

Banti and colleagues (Contribution 6) addressed a specific clinical problem: the hearing loss caused by cisplatin. Water-soluble polysaccharides from the cyanobacterium *Arthrospira platensis* raised the viability of cochlear HEI-OC1 cells in a dose-dependent manner and suppressed cisplatin-induced reactive oxygen species at 80 µg/mL, pointing to an antioxidant mechanism of protection. The authors also note the potential of these polysaccharides to serve as carriers for other otoprotective agents, which connects the mechanism to a plausible delivery strategy and to the formulation studies that follow. There is still no approved therapy to prevent cisplatin-induced hearing loss, so a protective natural product with a defined mechanism, even at the cell-model stage, targets an unmet clinical need.

## 4. Stabilization, Delivery, and Sustainable Application

The largest group of papers concerns the practical question of how to keep bioactive compounds intact and usable. Krystyjan and co-workers (Contribution 7) encapsulated extracts of *Sambucus nigra*, *Aronia melanocarpa*, and *Echinacea purpurea* in a psyllium/starch matrix, producing composites whose antioxidant capacity tracked total phenolic content closely (r > 0.94) and whose antimicrobial behavior depended on the extract used. The matrix retained the extracts in uniformly distributed structures with sizes of approximately 800–1500 nm, providing tunable thermal and surface properties, functioning as a suitable base for functional foods.

Legesse and colleagues (Contribution 8) compared maltodextrin and gum arabic as wall materials for the phenolics of *Verbascum sinaiticum*, an Ethiopian medicinal plant. Freeze-drying with maltodextrin yielded favorable outcomes, including an encapsulation efficiency of 81.31% and retention of 71.84% of phenolic content over 32 days of storage, along with controlled release under simulated gastric and thermal conditions. Encapsulation also masked the off-flavors that had limited the extract’s use, so the study is a clear example of formulation turning an otherwise impractical ingredient into a usable one.

Lapčík and colleagues (Contribution 9) took a different delivery route, using high-pressure homogenization to load natural dyes—raspberry anthocyanins, copper chlorophyllins, and β-carotene—into lecithin liposomes stabilized with carboxymethylcellulose. The resulting dispersions were physically stable; median particle sizes for raspberry anthocyanin and copper chlorophyllin systems were approximately 200 nm, whereas the β-carotene system presented a broader size distribution (165–405 nm). System zeta potentials were around −30 mV, with the highest encapsulation efficiency observed for anthocyanins (36.17% to 84.61%). By optimizing the formulation through a central composite design, the authors give food technologists a workable method for stabilizing colorants that would otherwise degrade quickly.

The collection’s single review, by Sabir and co-workers (Contribution 10), draws these application themes together. It surveys how polysaccharides from plants, fungi, marine organisms, animals, and microbes act as antioxidant, antimicrobial, and barrier-forming agents in edible films, coatings, and encapsulation systems that extend the shelf life of fruit, vegetables, meat, dairy, and bakery products. The review does not gloss over the obstacles—variable stability, sensory effects, and regulatory hurdles—that stand between laboratory results and commercial use. Placed alongside the experimental encapsulation studies, it supplies the broader context in which they sit. It also makes the point that protection is only half the story: the same carriers can govern where and how quickly a compound is released, which matters as much for a functional food as for a medicine.

## 5. Conclusions and Outlook

Read together, these ten contributions follow the path from finding a molecule to understanding and using it. Several rely on established methods, such as classical profiling, structure–activity work, and food-grade formulation, while others, in particular the combined mass spectrometry and chemical genomics platform and the cheminformatic dissection of essential-oil activity, point toward integrated, computation-assisted workflows that are reshaping the field. The gaps are worth noting. Direct target identification by chemoproteomics, the use of organoid models, and the microbial production of scarce metabolites remain uncommon in practice across much of natural product research, and they mark obvious directions for a third edition of this Special Issue. The therapeutic threads running through the collection, from antifungal and antimicrobial resistance to oxidative-stress-driven disease and cancer, are areas where new chemical matter is still needed. Published open access, the collection is freely available to the food scientists, chemists, and pharmacologists whose problems it speaks to, and that cross-disciplinary reach is part of what a thematic issue can offer. We thank all of the authors for their work and the reviewers for their careful and constructive input, and we hope readers find useful material in this collection.
